# BoBER: web interface to the base of bioisosterically exchangeable replacements

**DOI:** 10.1186/s13321-017-0251-x

**Published:** 2017-12-12

**Authors:** Samo Lešnik, Blaž Škrlj, Nika Eržen, Urban Bren, Stanislav Gobec, Janez Konc, Dušanka Janežič

**Affiliations:** 1 National Institute of Chemistry, Hajdrihova 19, 1000 Ljubljana, Slovenia; 2 Jožef Stefan International Postgraduate School, Jamova 39, 1000 Ljubljana, Slovenia; 3 Faculty of Mathematics, Natural Sciences and Information Technologies, University of Primorska, Glagoljaška 8, 6000 Koper, Slovenia; 4 Faculty of Chemistry and Chemical Technology, University of Maribor, Smetanova 17, 2000 Maribor, Slovenia; 5 Faculty of Pharmacy, University of Ljubljana, Aškerčeva Cesta 7, 1000 Ljubljana, Slovenia

**Keywords:** ProBiS, Protein superimposition, Bioisosteres, Scaffold hopping, Ligand fragments

## Abstract

We describe a novel freely available web server Base of Bioisosterically Exchangeable Replacements (BoBER), which implements an interface to a database of bioisosteric and scaffold hopping replacements. Bioisosterism and scaffold hopping are key concepts in drug design and optimization, and can be defined as replacements of biologically active compound’s fragments with other fragments to improve activity, reduce toxicity, change bioavailability or to diversify the scaffold space. Our web server enables fast and user-friendly searches for bioisosteric and scaffold replacements which were obtained by mining the whole Protein Data Bank. The working of the web server is presented on an existing MurF inhibitor as example. BoBER web server enables medicinal chemists to quickly search for and get new and unique ideas about possible bioisosteric or scaffold hopping replacements that could be used to improve hit or lead drug-like compounds. 
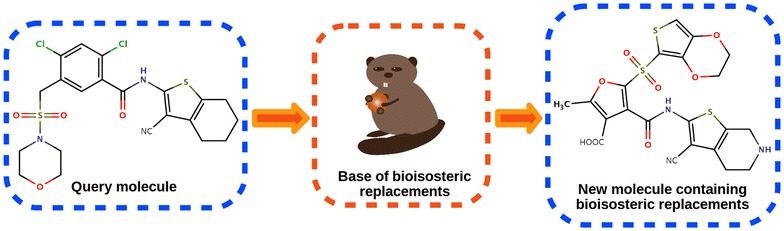

## Background

Bioisosterism and scaffold hopping are key concepts in the lead optimization stages of drug discovery [1, 2]. They can be defined as replacements of a part of a biologically active compound with a substructure that leads to a compound of the same or similar biological interaction. A bioisosteric replacement usually represents a functional group in a lead molecule that can be used in exchange of another functional group while the overall molecule retains similar non-covalent interactions towards a target. Bioisosteres are used to replace a functional group that is important for binding, but a new group in its place would improve the overall properties of a ligand, such as, lessen side-effects, improve pharmacokinetic properties, improve selectivity, simplify synthetic routes, increase metabolic stability or help avoid patent related issues [3]. Moreover, scaffold hopping can be interpreted as a subclass of bioisosteric replacements, where a larger part of a ligand—the core scaffold—is replaced. This core scaffold is important due to formation of direct interactions with the target or alternatively, it may provide appropriate scaffolding that spatially arranges functional groups so that they are able to form the necessary interactions.

In the past, bioisosteric and scaffold hopping replacements were obtained experimentally using the trial-and-error approach, resulting in today’s extensive literature available to the medicinal chemistry community [4]. The collected data can be used to create extensive digitized databases of bioisosteric replacements. BIOSTER [5], for instance, contains bioisosteric transformations collected from literature published in the last 40 years. ChEMBL [6] is a public domain database of over 1.5 million small molecules paired with associated bioactivity data mined from medicinal chemistry literature. The database enables identification of experimentally observed molecular substructures that exhibit bioisosteric characteristics. Based on these data, the Matched Molecular Pair (MMP) approach [7] enables the identification of molecules in ChEMBL that differ only in one functional group. This allows for the analysis of potential changes in biological properties that may be affected by such transformation. The MMP has been made freely available for non-commercial use on-line as the Swiss-Bioisostere database [8].

Rapidly growing freely available structural databases such as the Protein Data Bank (PDB) [9] offer another opportunity to obtain new bioisosteric and scaffold hopping replacements in a rigorous and automated way. Kennewell et al. [10] developed a method for comparison and superimposition of all *holo* proteins in the PDB based on protein backbone atoms, which allows ligands to be transposed between protein binding sites based on protein structure superimpositions. Fragments occupying the same geometric space are considered as potentially bioisosterically replaceable. Another method, KRIPO [11] quantifies similarities of binding site subpockets based on optimized pharmacophore based fingerprints, and enables both intra- and inter-family comparisons of proteins. Using this method, the complete PDB was converted into a database comprising of around 300,000 fingerprints of local binding sites together with their associated ligand fragments. The method enables the identification of bioisosteric replacements for ligand substructures based on local binding site similarities independently from the protein sequence or overall protein folding. Khashan [12] developed FragVLib, a virtual library of fragments which enables finding bioisosteric replacements based on a subgraph matching tool that identifies similar binding pockets according to their 3D structures and chemical composition. Further, sc-PDB-Frag [13] is an approach that considers bioisosteric searches with no a priori knowledge of either ligand (fragment) or protein (binding site) similarities. This can be achieved by converting protein–ligand interaction patterns to 1D or 3D graphs. Bioisosteres are then defined as any pair of ligands that share similar interaction patterns with their native target protein. Because the selection is directly based on protein–ligand interactions it does not require any pairwise similarity calculation between either ligands or binding sites. To extend the repertoire of methods for obtaining bioisosteric and scaffold replacements, we developed a freely available pre-calculated database of bioisostere replaceable fragments obtained with a rigorous all-against-all PDB local binding site alignments. Additionally, we developed a corresponding web interface, which enables easy acquisition of appropriate fragment replacements.

In this work we present Base of Bioisosterically Exchangeable Replacements (BoBER), a new web server for identification of bioisosteric and scaffold hopping replacements based on our PDB mining approach [14]. In this approach, bioisosteric replacements are identified using local binding site alignment algorithm ProBiS [15–19], which enables identification of locally similar binding sites irrespective of proteins’ folds or amino acid sequences. It seeks for similar local spatial arrangements of physico-chemically similar surface functional groups in binding sites, enabling the detection of replaceable fragment pairs between distantly related protein structures. ProBiS was used to superimpose *holo* binding sites from the entire PDB, and pairs of bioisosterically replaceable fragments were collected in the BoBER database [14]. The advantage of our method, which takes into account local neighborhood of fragments, is that it enables the distinction between different binding pockets in proteins with similar overall sequence identity, while recognizing similar binding pockets in proteins with very different sequences. This assures that identified bioisosteres will form similar interactions in the new environment of a possibly unrelated protein, while reducing the number of obtained bioisosteres that would not be able to form an appropriate interaction pattern with the protein’s binding site. BoBER web server is interactive and freely available at http://bober.insilab.org, and will benefit medicinal chemists in the lead optimization stage of the drug design process. The web server was tested in the Chrome and Firefox web browsers.

## Design and implementation

### Generation of database of bioisosteric replacements

As described previously [14], the process of bioisosteric replacement identification is started by using ProBiS to superimpose *holo* protein structures from the PDB. In this process, small-molecule ligand binding sites that have similar three-dimensional amino-acid arrangements are superimposed. We consider two binding sites as similar if the Z-score of their superimposition is equal or larger than two. Z-score indicates how many standard deviations the current alignment score differs from the average Z-score, calculated from all binding sites alignments over the entire PDB [19]. The co-crystallized ligands are subsequently transposed between similar superimposed binding sites based on the translation and rotation matrices obtained with binding site superimpositions. These matrices represent the linear transformation of the similar binding site atoms’ coordinates towards the superimposition on the query binding site coordinates. The transformed ligands are then fragmented to more basic substructures, such as individual rings and functional groups, which are able to form non-covalent interactions with the target. Fragment pairs exhibiting high spatial overlap measured by Hausdorff distance (HD) are considered as bioisosteric or scaffold replacements. We used this measure due to its computational efficiency, as many fragment pair overlaps had to be evaluated. HD is defined as the maximum of all the distances from a point in one set to the closest point in the other set. In our case the two sets represent the van der Waals surfaces of the fragments, therefore the HD distance between fragments A and B is defined as:

$$HD\left( {A,B} \right) = max\left\{ {oHD\left( {A,B} \right),oHD\left( {B,A} \right)} \right\}$$

where oHD(A, B) is the one-sided HD distance between fragments A and B being the maximum of all the distances from a point in fragment A to the closest point in fragment B.

### BoBER web server

The BoBER web server enables intuitive and fast searching of bioisosteric replacements for drug-like molecules in the previously prepared database of bioisosteric fragments. The web interface enables the user to broaden the search for bioisosteric replacements by implementing Rules 1–5 also described previously [14], which rely on the concept of *join* and *core* atoms. These are defined as: *join* atoms are atoms at which the rotatable covalent bonds are broken during the fragmenting process; *core* atoms are all atoms that are not *join* atoms. Rule 1 broadens the chemical space in which replacements are sought for, as it permits fragments with similar *join* atoms e.g. atoms with the same hybridization type (in addition to fragments with exact same *join* atoms) to be considered replaceable. The rule can be turned on or off using the *Loose filtering* or *Rigorous filtering* respectively, before the initiation of the screening procedure (see Table 1). Alternatively, this option can be chosen separately from others using the *Interchangeable join atom types* radio button found in the *Custom options* menu. Rule 2 which allows the conversion of *join* atoms to *core* atoms in bioisosteric fragments if the *join* atom (from the bioisosteric fragment) has a corresponding overlapping *join* atom (on the query fragment) was found to be rarely applicable and has been omitted from the interface. Consequently, all *join* atoms that are not part of the specified (selected) pair are always ignored. Rules 3 and 4 can be used together as part of the *Loose filtering* radio button selection. The combination of Rule 3 and 4 can also be implemented separately from other options using the *Use structures with common core as queries* radio button within the *Custom options*. Rule 3 initially removes all the *join* atoms from the query fragment and thus all fragments from the database that exhibit the same *core* structure (structure independent of *join* atoms) as the query are sought for. For example, if fragment *a* is the original query, and fragment *b* has the same *core* structure, then bioisosteric fragments of both *a* and *b* will be retrieved. Using Rule 3 we disregard *join* atoms which define how the bioisosteric structure should be reconnected back to the original molecule. Therefore, a new *join* atom is defined on the bioisosteric structure, by mapping the selected *join* atom of the query to the bioisosteric structure as defined by Rule 4. In cases when a bioisosteric fragment is reconnected to the original molecule with two or more *join* atoms, e.g., when replacing central fragment with two bonds to the rest of the molecule, BoBER enables the definition of only one pair of reconnecting *join* atoms (when *Use structures with common core as queries* option is not used). Any other bonds are formed between pairs of bioisostere fragment atoms and query fragments atoms which are closest together and where both of them are still available for bonding based on their valance number; e.g. a bond to a carbon atom can only be formed if it currently has less than four bonds, where bonds to hydrogen atoms are ignored. In ([14]) we also define Rule 5, which states that Rules 1–4 can be sequentially combined, and which is implicitly used in the BoBER web server.

**Table 1:** The details of the Loose and Rigorous filtering options

	Loose filtering	Rigorous filtering
Bioisosteric fragment pair is from the same SCOP family	Both (inter- and intra-family)	Intra-family
Interchangeability of similar join atom types	Irrelevant^a^	Non-interchangeable join atom types
Consideration of join atoms	Use structures with common core as queries (ignore join atoms)	Use specific structure as query (consider join atoms)

^a^Join atoms are ignored when the option *Use structures with common core as queries* is enabled

### Input query

Three input options to search for bioisosteric or scaffold hopping replacement fragments are available. The first provides the JavaScript Molecular Editor (JSME) [20] to enter a molecule, for example a drug structure, on which bioisosteric replacements are to be performed. After clicking the submit button, BoBER fragments the input structure, fragments of which will be presented after a few moments. A query fragment can then be selected to search for its bioisosteric replacements as described below.

The second query option is to *Draw* the *core structure* (without *join* atoms) of the fragment using the JSME. BoBER will output query fragments contained within the BoBER database that have the same substructure present within its *core* structure. Again, this fragment can be selected to find its bioisoteric replacements.

The third option is to specify the properties for the query fragment, for which we wish to find replacements. These properties include simple descriptors, such as the number of heavy atoms a fragment contains, the number of potential hydrogen bond donors and acceptors, number of atoms in rings or the number of *core* and/or *join* atoms.

In all three options, upon clicking the *Submit query* button, a *Fragment selection* panel is displayed, containing query fragments meeting the chosen criteria. A query fragment for which we wish to display its possible replacements can be selected, after which the user can define the *Overall Hausdorff distance* cutoff, which defines the extent of overlap between all of the database fragment atoms and the query ones. Lower HD requires better spatial overlap of corresponding ligand atoms in the superimposed binding sites. By visual inspection of a large number of pairs, we set the default value of *Overall HD* to 1.50 Å, which is loosely the maximum at which fragments can still be considered as replaceable. Fragments can also be filtered based on the superimposed proteins’ SCOP families [21]. Choosing the *Rigorous filtering* radio button selects the *Intra*-*family* option which limits the best fragment pairs (lowest HD) to the part of the database obtained from superimposed protein structures belonging to the same SCOP family. The *Inter*-*family* option, available in the *Custom options*, outputs fragments that originate from proteins that are of different SCOP families or when one or both of the protein families are unspecified. When selecting *Both* we get the best fragment pairs independently of this criteria. The *Both* option is selected as default when using the *Loose filtering* radio button. The protein-family related criteria refers to the superimposed proteins within the BoBER database independent of the target family to which we want our changed ligand to bind, as the current version of BoBER does not yet support this specification.

### Output of replaceable fragments

After query fragment selection and submission of HD-based criteria, a new *Results* tab opens. This tab contains a table, which displays the 2D structures of the query and reference fragment pairs and their corresponding HDs. *Join* atoms that are in spatial overlap between two fragments, that is corresponding *join* atoms, are shown with the same highlight colors. When using the *Use structures with common core as queries option* (part of the *Loose filtering* option), the bioisosteric fragments are shown reduced to their *core* structure. If the *Use specific structure as query* radio button has been selected, then the user can sort the bioisosteric pairs based on three different HD values (*Overall*, *Core* or *H*-*bonding* HD) in ascending or descending order. When *Use structures with common core as queries* is selected, the sorting can be done only based on the *Core HD* as the other HDs that are based on *join* atoms are not relevant in this case. By clicking on the structure image of a fragment, a new tab opens in the browser with the PDB web page of the protein–ligand complex from which the fragment was obtained.

## Usage example

**Fig. 1:**
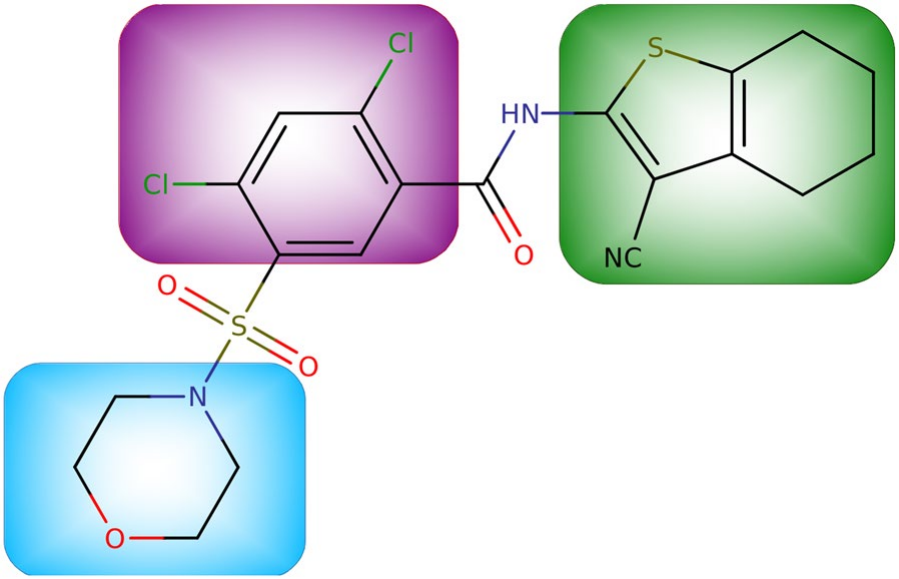
MurF enzyme inhibitor. Fragments we wish to replace are highlighted with blue (morpholine), purple (dichlorobenzene) and green (benzothiophene)

Use of BoBER is presented on an inhibitor of the MurF (UDP-N-acetylmuramoyl-tripeptide–D-alanyl-d-alanine ligase) bacterial enzyme (Fig. 1). MurF is a muramyl ligase, an intracellular, ATP-dependent enzyme that catalyzes the final intracellular peptidoglycan biosynthesis step [22–25]. As MurF has no human counterpart and the inhibition of peptidoglycan biosynthesis leads to a reduced rate of bacterial cell reproduction it is an appropriate target for the development of antibacterial drugs. The inhibitor used here is a sulfonamide type inhibitor of MurF discovered by Abbot Laboratories in 2006 [26]. However, it was found that this inhibitor lacks antibacterial activity, probably due to its poor cell permeability. Based on the findings in Ref. [24], we used BoBER to obtain bioisosteric replacements for the ring fragments of this inhibitor, which could potentially lead to its improved antibacterial activity.

**Fig. 2:**
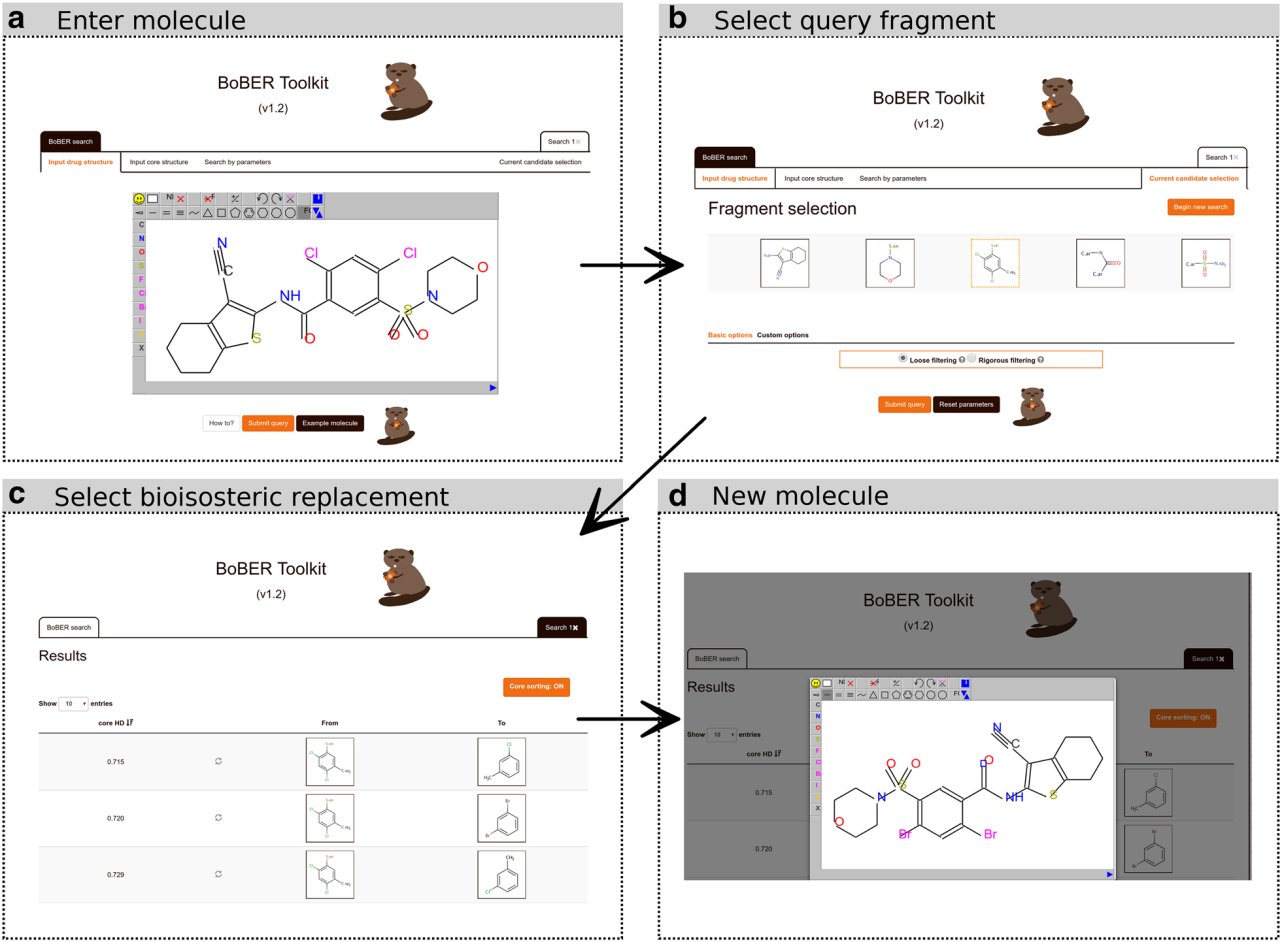
Workflow of the BoBER web server usage

The inhibitor structure was entered into the web server using the first, that is the *Input drug structure*, input option (Fig. 2a). Five fragments were obtained after fragmentation, including each of the three query cyclic fragments that we wished to replace (Fig. 2b). Using the default HDs, and clicking the *Submit query* button, initiated the database to be searched for bioisosterically replaceable fragments; after a few moments the output page was displayed (Fig. 2c). In case of when the initial molecule is drawn using the *Input drug structure* option, BoBER also enables the exchange of the original fragment with the selected bioisosteric fragment by clicking on the » refresh « glyphicon (two arrows in a loop) left of the bioisosteric pairs images. This action opens a dropdown menu of possible *join* atoms which can be used to reconnect the bioisosteric fragment to the original structure in place of the original fragment (Fig. 2d). When using *Loose filtering or Use structures with common core as queries* option, only the *join* atoms of the query are shown, as *join* atoms on the potential bioisosteric structures have no meaning. The whole structure with bioisosteric replacements can then be displayed in the JSME molecular editor. By right-clicking on the editor’s window the obtained bioisosteric structure of the compound can be exported to different chemical file formats, such as SMILES, MOL or InChI for use in downstream operations.

In Table 2 are examples of bioisosteric replacements of the three cyclic fragments that were found to be of interest by visual examination. We chose bioisosteric fragments that were not trivial or too similar to the original fragment. With replacement **1** we used the *Rigorous filtering* option, where the *Interchangeable join atom types* radio button was additionally selected within the *Custom options* menu as without this, no replacements could be found. Due to the latter option the *N.sp2 join* atom could be exchanged with *N.pl*, therefore making the replacement possible. For replacements **2** and **3** we used the *Loose filtering option* to obtain suitable and nontrivial replacements, resulting in only the *core* structure of the original fragment being identical to the query fragments, while *join* atoms differed as they were ignored in the database screening procedure. Bioisosteric fragments are presented as a list divided into pages of 10 fragments each, and are sorted according to their overlap with the query fragment measured by the Hausdorff distance. The structurally diverse fragments therefore tend to be at the bottom of the list, e.g., replacement **2** is on page 18 and replacement **3** on page 8, which does not indicate that they are less active than those at the top of the list. Because each bioisosteric fragment pair is obtained from a pair of similar binding sites, the activity of a bioisosteric fragment depends on this similarity as well as on its overlap with the query fragment. Bioisosteric fragments can be found multiple times in the results list with different HDs, e.g., the benzene ring when the query is fragment **2** (morpholine). The reason for this is that the same bioisosteric fragment can be obtained from multiple different superimposed protein structures. This can be seen by clicking on the structure image of morpholine or benzene, which opens the PDB page of their corresponding proteins from which these fragments were obtained. For example, the first pair (HD of 0.767) is from proteins with PDB codes 4u8z (morpholine) and 3f66 (benzene), while the second (HD of 0.794) is from 4yff (morpholine) and 3pxq (benzene). Therefore, as they originate from different protein superimpositions, they have different Hausdorff distances.

**Table 2:** Possible structures with bioisosteric replacements found using BoBER

Replacement number	Original fragment	Bioisostere found by BoBER	Similar bioisostere found by SwissBioisostere
**1**			
**2**			
**3**			No similar

Original join atoms are shown, however in **2** and **3** they were ignored during the procedure, as per usage of the Loose filtering option

**Fig. 3:**
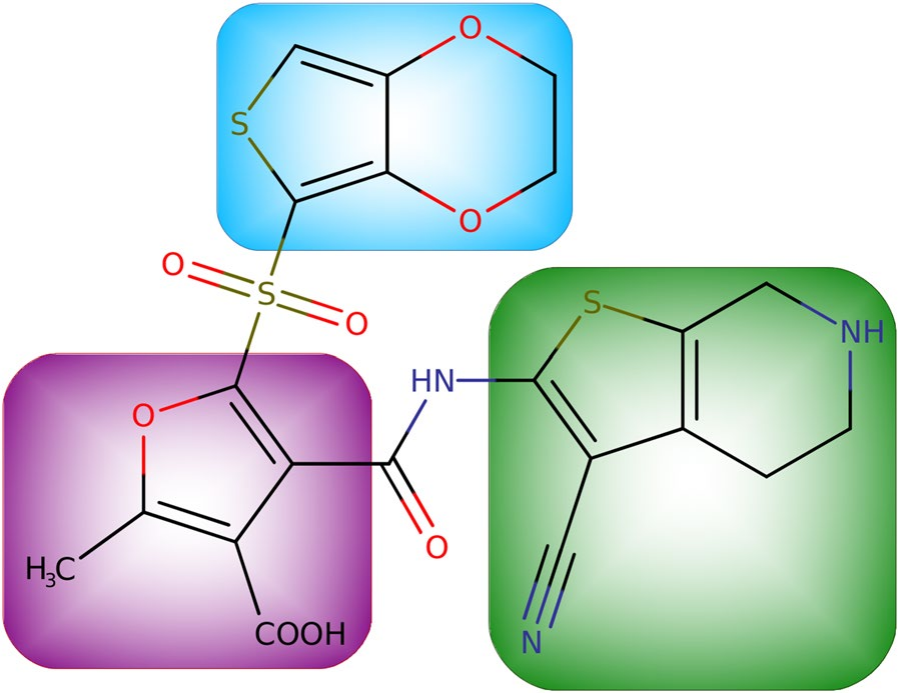
A bioisosteric version of MurF enzyme inhibitor from Fig. 1. All the replacements from Table 2 were performed sequentially, one fragment at a time. Each time, the SMILES of the resulting bioisosteric compound with one of the fragments replaced was copied to a new search tab, and the procedure was repeated for the next fragment. Bioisosteric fragments are highlighted using the same colors as the original fragments in Fig. 1 which are replaced

The final structure of bioisosterically replaced new MurF inhibitor, using example replacements from Table 2, is presented in Fig. 3. We were therefore able to obtain a unique structure with BoBER that was not previously described in the mentioned baseline article [24]. In Ref. [14] we performed a docking study with the original and bioisosteric inhibitor of the butyrylcholinesterase enzyme. Both docking scores were within the standard error of the docking program suggesting similar binding affinities. The BoBER web server is primarily an idea generator for medicinal chemists that enables trying different fragment options in the drug optimization phase to possibly improve pharmacokinetics and selectivity and also to diversify the compounds’ scaffolds.

### Comparison of BoBER performance

We compared BoBER with SwissBioisostere database [8], which is another freely available tool for obtaining bioisosteric replacements. The two approaches differ significantly, therefore we expected different results. We queried the SwissBioisostere database with the three ring fragments (Table 2) previously used in BoBER, where we replaced join atoms with the R-groups indicating attachment points. For fragment **1**, we obtained six suggested replacements, none of which exactly matched the bioisosteric fragment obtained with BoBER. However, the cyclopentathiophene-carbonitrile fragment suggested by the SwissBioisostere is similar to the fragment suggested by BoBER (first row, Table 2). For fragment **2**, we obtained many potential replacements using both servers, and several similar bioisosteres were found. For example, compare bioisosteres of fragment **2** obtained by BoBER and SwissBioisostere (second row, Table 2), where a thiophene in BoBER fragment is replaced with a benzene. It is well known that thiophene is a bioisosteric replacement for benzene. Finally, no similar bioisostere was found among the results obtained with SwissBioisostere for BoBER’s bioisostere of fragment **3** in which an acidic moiety is bound to a furan ring. There seems to be some overlap between the bioisosteres found by BoBER and SwissBioisostere. BoBER also finds different replacements that might not have been included in SwissBioisostere as of yet.

## Conclusion

We developed a new web server BoBER that enables the prediction of bioisosteric replacements given a query fragment or query small molecule as input based on our knowledge-based method that uses binding sites superimposition to identify possible bioisosteric and scaffold hopping replacements from existing ligands. The predicted bioisosteric replacements are obtained after the ProBiS-based superimposition of existing PDB crystal *holo* protein structures, which makes us confident that a significant proportion of newly generated compounds will retain activity. The database of bioisosteric pairs obtained with this method is implemented in a freely available web server BoBER, which enables medicinal chemists to quickly search and get new and unique ideas about possible bioisosteric or scaffold hoping replacements that could be used to improve hit or lead structures. We showed how the BoBER web server could be used on an inhibitor of MurF enzyme. In the future, the BoBER approach will be implemented in the ligand-based virtual screening software LiSiCA [27] to enable searching databases for similar ligands not only on the basis of atom type similarity but also based on possible bioisosteric or scaffold hopping replacements.
